# Polymer-assisted synthesis of ternary magnetic graphene oxide nanocomposite for the adsorptive removal of Cr(VI) and Pb(II) ions

**DOI:** 10.1016/j.heliyon.2024.e35204

**Published:** 2024-07-25

**Authors:** Qaisar Manzoor, Muhammad A. Farrukh, Muhammad T. Qamar, Arfaa Sajid, Samar A. Aldossari, A. Manikandan, Munawar Iqbal

**Affiliations:** a Department of Chemistry, Forman Christian College (A Chartered University), Ferozepur Road, Lahore, 54600, Pakistan; b Department of Chemistry, The University of Lahore, Lahore, Pakistan; c Department of Basic and Applied Chemistry, Faculty of Science and Technology, University of Central Punjab, Lahore, Pakistan; d Department of Chemistry, College of Science, King Saud University, P. O. Box 2455, Riyadh, 11451, Saudi Arabia; e Department of Chemistry, Centre for Material Chemistry, Karpagam Academy of Higher Education, Coimbatore, 641 021, Tamil Nadu, India; f School of Chemistry, University of the Punjab, Lahore, 54590, Pakistan

**Keywords:** Graphene oxide, Polymers, Chitosan, Polyaniline, Fe_3_O_4_, Nanocomposite, Adsorption, RSM-BBD

## Abstract

The presence of chromium [Cr(VI)] and lead [Pb(II)] ions in the water bodies have adverse effects on humans and aquatic life. Graphene oxide-based magnetic nanocomposites synthesized in the presence of chitosan (mGO/CS) or polyaniline (mGO/PA) as potential adsorbents for the removal of Cr(VI) and Pb(II) ions. The FTIR (Fourier transform infrared spectroscopy), EDX (Energy dispersive X-ray), XRD (X-ray diffraction) and SEM (Scanning electron microscopy) were employed to investigate the chemical composition, structural, elemental analysis, crystalline size and morphology of the nanocomposites. The FTIR results confirmed the synthesis of the nanocomposites by detecting peaks of specific functional groups. The average crystallite sizes of the mGO, mGO/CS, and mGO/PA nanocomposites were 17, 25, and 23 (nm), respectively, as determined by the Debye-Scherrer equation from the XRD data. Batch adsorption experiments were conducted for Pb(II) and Cr(VI) removal by varying the variables like pH, concentration of metal ions and contact time. The Box Behnken design (BBD) was used to optimize the adsorption parameters. Under the optimum conditions, mGO/CS and mGO/PA showed maximum removal percentages (%R) of 92.36 and 98.7 for Pb(II), and 85.25 and 93.08 for Cr(VI), respectively. The adsorption capacities were 110.84 and 118.44 mg/g for Pb(II), and 87.74 and 111.7 mg/g for Cr(VI) were obtained for mGO/CS and mGO/PA, respectively. The pseudo-second-order kinetic model and Langmuir isotherm fitted well to the experimental data and explain the adsorption mechanism of the nanocomposite materials for both metal ions.

## 1 Introduction

Water pollution is a critical environmental crisis driven by unchecked urbanization and the rapid expansion of industries worldwide. Industrial effluent discharge, urban runoff, and improper waste disposal contribute to the contamination of rivers, lakes, and groundwater with hazardous substances, such as lead, mercury, cadmium, and chromium. Toxic heavy metals can accumulate in water bodies, sediments, and aquatic organisms, posing a significant threat to aquatic ecosystems [1,2]. Owing to the persistent and non-biodegradable nature of these contaminants, polluted water frequently infiltrates the human water supply, causing adverse health effects even at low concentrations [3]. To protect aquatic ecosystems and reduce public health risks, a comprehensive approach that includes pollution prevention, remediation measures and the promotion of eco-friendly alternatives in industrial processes is required. The impact of heavy metal water pollution can be reduced by raising awareness and implementing responsible practices to protect both the environment and public health for current and future generations [4,5].

Among heavy metals, Pb(II) has a significant threat to both the environment and human health, due to its toxic nature and tendency to bioaccumulation in the food chain. Pb(II) ions enter into the natural water bodies as a result of various industrial wastewater discharges e.g. paper, electroplating, metallurgical finishing, storage-battery, dyeing, mining and automotive industries [6,7]. The major sources of Cr(VI) ions contamination are textiles, paints, pigments, pulp, metallurgical processes, and tanning industries. The Cr(VI) ion toxicity depends on their mobility and solubility within the environment. The Cr (III) ions have low water solubility as compared to the Cr(VI) ions, which reduced its toxicity as compared to the Cr(VI) ions [8]. Therefore, for the protection of the environment and human health, industries must adopt advanced water treatment technologies and employ purification processes to eliminate or significantly reduce the heavy metal concentration in their effluents before they enter the water system [9].

There are different techniques reported for eliminating toxic metal ions including chemical precipitation, nanofiltration, ultra-filtration, reverse osmosis, ion exchange and coagulation. However, these have several limitations for example low removal efficiency, sludge production and high operational cost. In contrast, the adsorption method showed more advantages such as lower energy consumption, easy procedures, cost-effective and good % removal efficiency [10].

Graphene oxide (GO) is a hydrophilic derivative of graphene, which has a planar arrangement of carbon atoms forming a hexagonal lattice structure, consisting of a two-dimensional carbon lattice decorated with numerous functional groups (oxygen-containing), such as hydroxyl (-OH), epoxied (-O-), and carboxyl (-COOH). The distribution of oxygen functional groups is not uniform, so resulting in a heterogeneous structure. This property allows it to disperse readily in water and other polar solvents and makes it highly reactive. It can be easily functionalized further or used as a surface for the attachment of various molecules, making it versatile for various applications. GO has a high surface area and several functional groups, which make it an excellent adsorbent for various substances, including heavy metals, organic pollutants and biomolecules [[11], [12], [13], [14]].

Magnetic nanoparticles (Fe_3_O_4_) promising alternative to traditional adsorbents in surface adsorption processes. These nanoparticles possess excellent dispersibility, high surface area, and high surface-to-volume ratio. Their simple synthesis and versatility for surface modification make them effective adsorbents for various water purification applications [15]. Fe_3_O_4_ nanoparticles present numerous advantages, such as minimal toxicity, simple synthesis, affordability, strong magnetic characteristics, effective adsorption capability and rapid adsorption rates [16,17]. However, Fe_3_O_4_ nanoparticles are rarely used alone in surface adsorption processes due to their limited selectivity. To address this limitation, they are often modified with different nanomaterials. These modifications are designed to improve their surface properties and selectivity, thereby enhancing their efficiency and effectiveness for targeted adsorption applications. GO nanocomposites have significant attention in the adsorption process due to their excellent mechanical strength, high surface area, and tunable surface chemistry, making them highly effective for various adsorption applications. Their versatility is further enhanced when combined with polymers such as chitosan and polyaniline, which contribute additional functional groups and improve adsorption efficiency. Consequently, GO nanocomposites are important for eliminating environmental pollutants and presenting a promising solution for advanced water purification and environmental remediation efforts [18,19]. According to the literature, different polymers are applied for the functionalization of GO/metal oxide for good dispersion, high interaction between the components, enhanced mechanical properties and better performance of the final material. The mGO/chitosan nanocomposite to remove the Zn (II) and Hg (II) ions showed a removal of 96.73 % removal of Zn (II) ions within 38 min and 86.96 % removal of Hg (II) ions within 8 min [20,21]. To enhance the surface chemistry of GO, in this research chitosan (CS) and polyaniline (PA) were employed in the polymer-assisted synthesis of mGO nanocomposite. Initially, GO was synthesized using the modified Hummers' method, followed by surface modification (functionalization) with each of the above-mentioned polymers. Afterward, Fe_3_O_4_ nanoparticles were attached to the modified surface to further enhance the adsorption properties of the nanocomposite.

Various statistical models have been employed across different fields to optimize the process. Among these, Response Surface Methodology (RSM) is a widely used statistical model in experimental design. RSM helps evaluate the effects of independent variables on response outcomes and predicts the optimal response. Importantly, RSM reduces chemical consumption and simplifies the experiments needed to determine the best conditions for the variables [22]. The present study aimed to remove toxic metal ions Cr(VI) and Pb(II) from water samples using a polymer-assisted synthesis of GO/Fe_3_O_4_ (mGO/polymer) nanocomposite as a highly efficient adsorbent. Different adsorption parameters, including the concentration of metal ions, metal solution pH and contact time were examined for optimum purpose. The optimization of all these adsorption parameters was carried out by BBD-RSM software. To the best of our knowledge and based on thorough literature research, this approach has not been previously employed for optimizing for Pb(II) and Cr(VI) ions by mGO/polymer nanocomposites. Hence, the application of BBD-RSM for optimization in work presents a novel contribution to this field.

## 2 Materials and methods

### 2.1 Chemicals

The chemicals utilized in the experimental procedures were of analytical grade and utilized without any further purification. Graphite powder, chitosan, aniline, ammonium persulfate (APS), lead acetate, sodium hydroxide, potassium dichromate, sulfuric acids, hydrogen peroxide, acetic acid, iron (II) acetate (Fe(CH₃COO)₂), hydrochloric acid, iron (III) nitrate nonahydrate (Fe(NO_3_)_3_.9H_2_O) and potassium permanganate were obtained from Merck and Sigma Aldrich.

### 2.2 Synthesis of GO

**Fig. 1:**
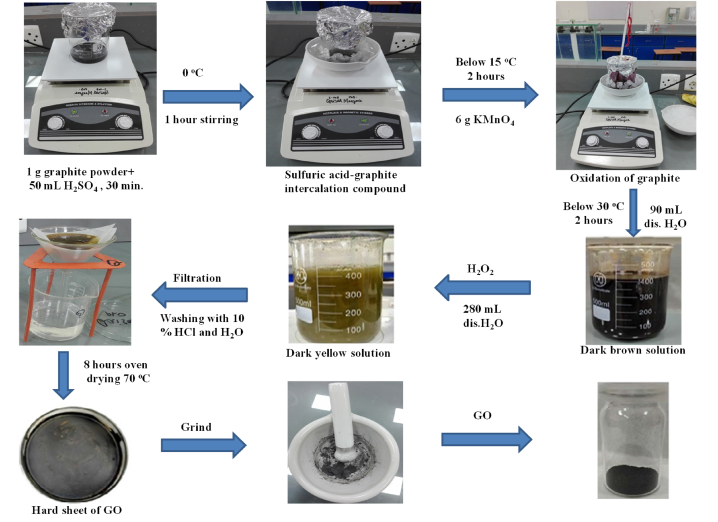
Schematic representation for the synthesis of GO from graphite powder.

Graphite powder was used for the preparation of GO using a modified version of the Hummers' method as reported in earlier studies [23,24]. In brief, 1 g graphite powder with 50 mL of conc. H_2_SO_4_ was stirred for 30 min at 25 °C. The mixture was then chilled at 0 °C using an ice bath and stirred further for 60 min. Later, 6 g of KMnO_4_ was added while ensuring the temperature remained below 15 °C. After continuous 2 h stirring, 90 mL of distilled water was introduced slowly and the temperature was maintained under 30°C for another 2 h stirring. The mixture was further diluted by adding 280 mL of water, and then 6 mL of H_2_O_2_ was added to the mixture with vigorous stirring. The development of a vibrant yellow hue indicated the successful formation of GO. After filtration, the product was washed with 10 % HCl followed by distilled water and finally, dried it overnight at 70 °C (Fig. 1).

### 2.3 Fe_3_O_4_ nanoparticle synthesis

Magnetite Fe_3_O_4_ NPs were prepared using the co-precipitation method. 50 mM iron acetate (Fe (CH₃COO) ₂) and 100 mM solution of Fe(NO_3_)_3_·9H_2_O) at the mole ratio (Fe^3+^: Fe^2+^ = 2:1) was prepared and stirrer for 15 min 0.5M NaOH was added to above mention iron salt solution with the feed rate of 0.58 mL/5 min with continuous stirring till pH 10 [15]. Subsequently, the mixture was stirred and heated at 65 °C for 1 h. Black precipitates of Fe_3_O_4_ nanoparticles were obtained separated by employing an external magnetic field, and washed with distilled water until achieving a neutral pH to eliminate impurities. Moreover, the nanoparticles were dried in an oven for 3 h at 100 °C [25].

### 2.4 Synthesis of mGO/CS

The ternary nanocomposite mGO/CS was produced by the following method: Initially, 400 mg chitosan was mixed in 20 mL of a 1.8 % acetic acid solution, which was then subjected to 30 min of ultrasonication. The CS solution was mixed with the GO solution and the mixture was stirred for 2 h. Fe_3_O_4_ nanoparticle solution was added to the above mixture with constant stirring [26]. After filtration the product was washed out and dried in a drying oven; the final product was mGO/CS nanocomposite with ratios of 1:2:4 by mass respectively.

### 2.5 Synthesis of mGO/PA

The mGO/PA nanocomposite synthesized with the 1:2:4 ratios of masses respectively at room temperature. Briefly, 200 mg GO dissolved in 10 mL 1 M HCl solution and sonicated for 30 min 400 μL aniline was added to the GO solution with stirring and then sonicated the whole mixture for a further 30 min. Added 100 mg/10 mL metal oxide solution into the above mixture in 1 h with stirring. After that, 0.25M/20 mL solution of ammonium peroxydisulfate (APS) in 1 M HCl was added drop by drop to the previous suspension and polymerization was allowed to continue at 5 °C for 6 h. Finally, the resulting suspension was washed multiple times with double-distilled water until the supernatant reached a neutral pH. The mixture was then centrifuged and drying was done at 65 °C under a vacuum [27].

### 2.6 Characterization

A flame atomic absorption spectrometer (Varian 240 FS) was utilized with lead and chromium hollow-cathode lamps as a radiation source to detect the Pb(II) and Cr(VI) ions concentrations respectively. FTIR, Agilent technology (Cary 630) was used to appraise the functional groups with the range from 550 to 4000 cm^−1^. XRD, PANalytical Empyrean diffractometer, was used for the structural, phase identification and crystalline size determination. The examination of morphology was conducted by Hitachi S–3400N SEM and the elemental analysis was performed using the EDX system from Ametek.

### 2.7 Adsorption studies

In batch mode, the Pb(II) and Cr(VI) ions adsorption was examined utilizing fabricated ternary nanocomposites. The experiments involved varying the three suggested adsorption parameter values, as outlined in Table 1 for Pb(II) and Cr(VI) ions, respectively, at room temperature. The adsorption capacity at equilibrium qe (mg/g) and the percent removal (% R) were determined using equations (1), (2), respectively [28].

(1) $$q=\left\{{\left(C_{o}-C\right)}_{.\;}\left(\frac{V}{100}\right)\right\}/m$$

(2) $$\left(\%R\right)=\frac{C_{o}-C}{C_{o}}*100$$

Where C and C_o_ are the final and initial concentration of ions (mg/L) respectively, and V is the volume of metal solution (mL) while “m” is the nanocomposite mass in grams.

**Table 1:** Adsorption factors and their ranges for Pb(II) and Cr(VI) ions.

Factor	Unit	Lower limit	Upper limit
**A**: pH (Pb II) (Cr VI)	–	4 2	6 4
**B**: Metal concentration	mg/L	20	60
**C**: Contact time	min.	60	120

### 2.8 Response Surface Methodology (RSM)

The statistical program Design Expert 13 was used to plan and optimize the experiment. RSM is a collection of statistical, graphical, and mathematical methods used in problem modeling and analysis where multiple variables impact the response of interest [29]. Three adsorption parameters were optimized for the adsorption process using the Box-Behnken design (BBD). Based on Table 1 data [30], the lower and upper levels of three adsorption parameters and seventeen experiment runs were generated (Table 2, Table 3).

**Table 2:** RSM analysis for removal of Pb(II) ions using mGO/CS and mGO/PA nanocomposite.

				mGO/CS		mGO/PA	
Run	Factor A	Factor B	Factor C	Response 1	Response 2	Response 1	Response 2
	pH	Metal concentration	Time	Adsorption capacity (q_e_)	% Removal	Adsorption capacity (q_e_)	% Removal
		mg/L	min.	mg/g	%	mg/g	%
1	4	40	60	43.54	54.42	57.28	71.6
2	5	40	90	67.28	84.1	74.24	92.8
3	6	60	90	71.5	59.58	83.48	69.56
4	4	40	120	61.5	76.87	63.82	79.77
5	5	20	60	32.82	82.05	37.76	94.4
6	4	60	90	76.62	63.85	91.46	76.21
7	6	40	60	37.5	46.85	44.84	56.05
8	6	40	120	49.52	61.9	61.34	76.67
9	4	20	90	31.48	78.7	34.9	87.25
10	5	60	120	109.66	91.38	119.82	99.85
11	5	40	90	69.48	86.85	72.26	90.32
12	5	20	120	35.74	89.35	39.7	99.25
13	5	60	60	102.06	85.05	107.64	89.7
14	5	40	90	68.64	85.8	76.22	95.27
15	6	20	90	25.42	63.55	31.7	79.25
16	5	40	90	66.36	82.95	77.86	97.32
17	5	40	90	62.5	76.43	65.5	81.87

**Table 3:** RSM analysis for removal of Cr(VI) ions using mGO/CS and mGO/PA nanocomposite.

				mGO/CS		mGO/PA	
Run	Factor A	Factor B	Factor C	Response 1	Response 2	Response 1	Response 2
	pH	Metal concentration	Time	Adsorption capacity (q_e_)	% Removal	Adsorption capacity (q_e_)	% Removal
		mg/L	min.	mg/g	%	mg/g	%
1	4	40	60	32.9	41.13	39.66	49.57
2	3	20	60	23.28	58.2	29.28	73.2
3	3	40	90	41.48	51.85	48.64	60.8
4	4	60	90	61.38	51.15	65.54	54.62
5	3	60	60	75.28	62.73	81.54	67.95
6	2	20	90	32.86	82.15	36.62	91.55
7	3	40	90	42.56	53.2	51.5	64.37
8	3	20	120	27.24	68.1	34.22	85.55
9	4	40	120	46.82	58.52	47.26	59.08
10	3	60	120	91.24	76.03	96.32	80.27
11	2	60	90	105.16	87.63	115.62	96.35
12	2	40	60	55.26	69.07	67.28	84.1
13	3	40	90	45.5	56.87	53.34	66.67
14	3	40	90	46.78	58.47	55.12	68.9
15	4	20	90	21.68	54.2	24.22	60.55
16	3	40	90	46.44	58.05	54.26	67.83
17	2	40	120	68.66	85.82	76.44	95.55

## 3 Results and discussion

### 3.1 FTIR analysis

**Fig. 2:**
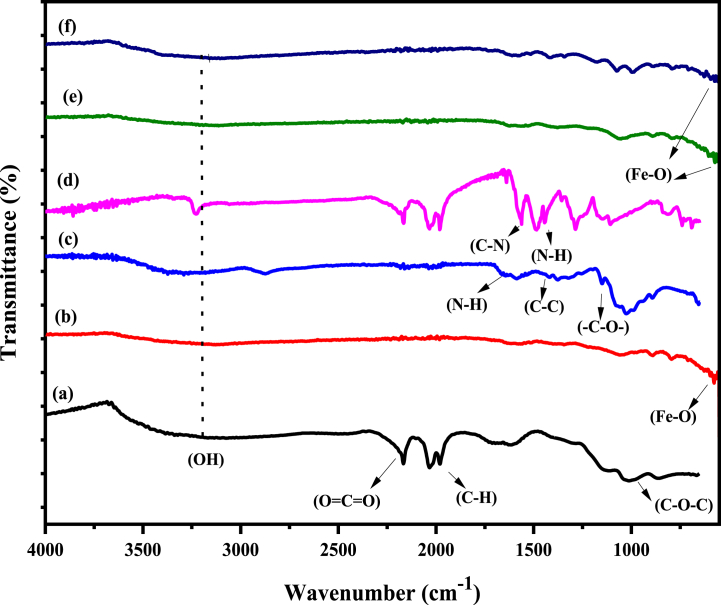
FTIR spectra of (a) GO (b) Fe_3_O_4_ (c) CS (d) PA (e) mGO/CS and (f) mGO/PA nanocomposites.

FTIR is a suitable method for analyzing the structure of synthesized nanocomposite and identifying the functional groups that are present on their surfaces. The FTIR spectrum for GO, Fe_3_O_4_, CS, PA, mGO/CS and mGO/PA nanocomposites are depicted in Fig. 2. FTIR spectrum of all nanomaterials showed a prominent and broad peak from 3200 to 3400 cm^−1^ due to the vibrational band of OH groups. In Fig. 2 (a) GO showed the peaks at 1630 cm^−1^ indicating the stretching vibration of carbonyl (C=O) and carboxylic group, the peak observed at 1050 cm^−1^ associated with the stretching vibrations of C–O in alkoxy and epoxy groups [31], Additionally, 1618 cm^−1^ peak attributed to the C=C stretching vibration while a peak at 1380 cm^−1^ was due to C–H bending vibration [32]. In Fig. 2 (c) a discrete peak at 1710 cm^−1^ was observed that corresponding to the N–H bending and amide (NH–C=O) stretching vibrations present in the chitosan, also appeared in Fig. 2 (e) which confirmed that CS is present in the final product of mGO/CS nanocomposite structure [31]. mGO/PA showed a peak at 3430 cm^−1^ attributed to the N–H peak while this peak is merged in the mGO/CS spectrum (Fig. 2(d)). Moreover, the peak detected at 1620 cm^−1^ related to the C=O stretching vibration in carboxyl groups that are present in the GO structure of all fabricated nanocomposites [13]. In mGO/PA a sharp peak observed at 1562 cm^−1^ was related to the C=C stretching of the quinoid ring (N = Q = N) while the peak at 1415 cm^−1^ was attributed to the C=C stretching vibrations of the benzenoid ring (N–B–N), these peaks are absent in mGO/CS spectra [33,34]. A blue shift is observed for the stretching vibrations of C-OC and values are increased as 1064 and 1072 for mGO/CS and mGO/PA respectively as shown in Fig. 2(e) and (f). These absorbance peaks signify the existence of oxygen-containing functional groups, formed during the oxidation process occurring on the surface of graphene oxide sheets derived from graphite. In Fig. 2(b)–(e) and (f) Fe_3_O_4_ nanoparticles show strong absorption peaks at 577 and 630 cm^−1^, attributed to stretching vibrations of Fe–O, which is similar to the FTIR spectrum of Fe_3_O_4_ nanoparticles reported in the previous studies [13,25].

### 3.2 XRD analysis

**Fig. 3:**
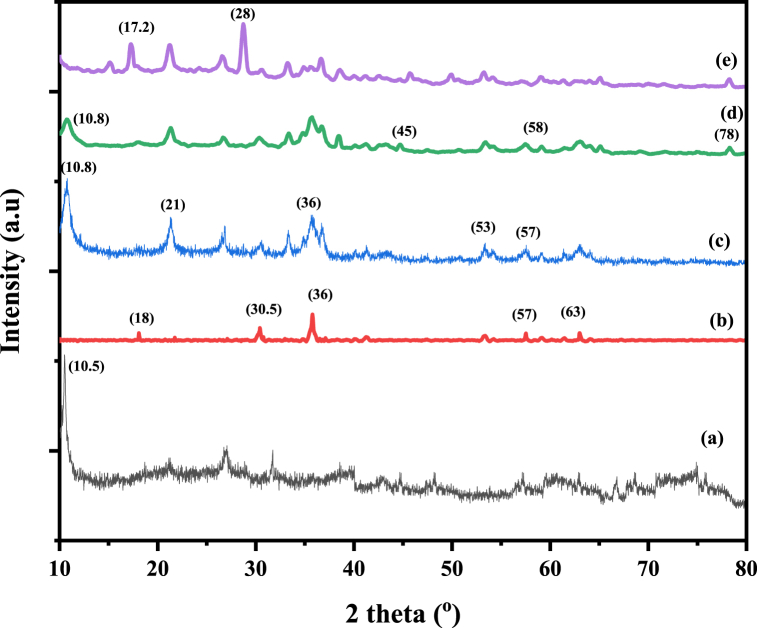
XRD patterns of (a) GO (b) Fe_3_O_4_ (c) mGO (d) mGO/CS and (e) mGO/PA nanocomposites.

XRD was used to examine the phase studies and structural properties of GO, Fe_3_O_4_, mGO, mGO/CS and mGO/PA nanocomposites. XRD patterns of all fabricated nanocomposites are represented in Fig. 3. Fig. 3 (a) showed the characteristic peak of GO at 2θ = 10.5^o^ that is also present in the XRD pattern of nanocomposite structures in Fig. 3(c) (d) and (e) spectra at 10.8^o^ which indicated the GO single-layered structure, previously reported [23,35] with literature (JCPDS 75–2078). The Fe_3_O_4_ XRD pattern in Fig. 3(b) showed seven distinct peaks at 18^o^, 30.5^o^. 36^o^, 43^o^, 53^o^, 57^o^ and 63^o^ [36]. XRD pattern of mGO peaks at 10.8^o^, 21.3^o^, 26.7^o^, 33.3^o^, 35.7^o^, 53.5^o^, 57.4^o^ and 63^o^ corresponding to the crystalline planes (002), (112), (200), (103), (204), (321) and (400) respectively. These peaks closely match with the standard XRD pattern typically observed for Fe_3_O_4_ nanoparticles in published literature and with the JCPDS 01-075-1609 and the crystal system is orthorhombic. XRD of mGO/CS showed all corresponding peaks of GO and Fe_3_O_4_ nanoparticles with the addition of CS peaks at 2θ = 45^o^, 58^o^, and 78^o^ which confirmed the successful grafting of CS on mGO nanomaterials [37,38]. While mGO/PA nanocomposite showed 2θ = 17.2^o^ and 28^o^ corresponding to (012) and (021) crystalline planes of polyaniline with a complete pattern of GO and Fe_3_O_4_ nanoparticles [33,39].

The D (average crystallite size) of mGO, mGO/CS and mGO/PA nanocomposites was determined by using the Debye-Scherrer equation (Eq. (3)) at various 2θ values for major peaks detected in XRD spectra.

(3) $$D=\frac{k\lambda }{\;\beta \;\cos\;\theta }$$

The crystalline size was measured in nm, in equation (3) “k” is the Scherrer constant (0.9), λ represents the X-rays wavelength (0.15406 nm) and “β” is the FWHM for each peak at a particular angle. The average crystallite sizes (D) of mGO, mGO/CS and mGO/PA nanocomposites were to be 17, 25 and 23, nm respectively.

### 3.3 EDX and SEM analysis

**Fig. 4:**
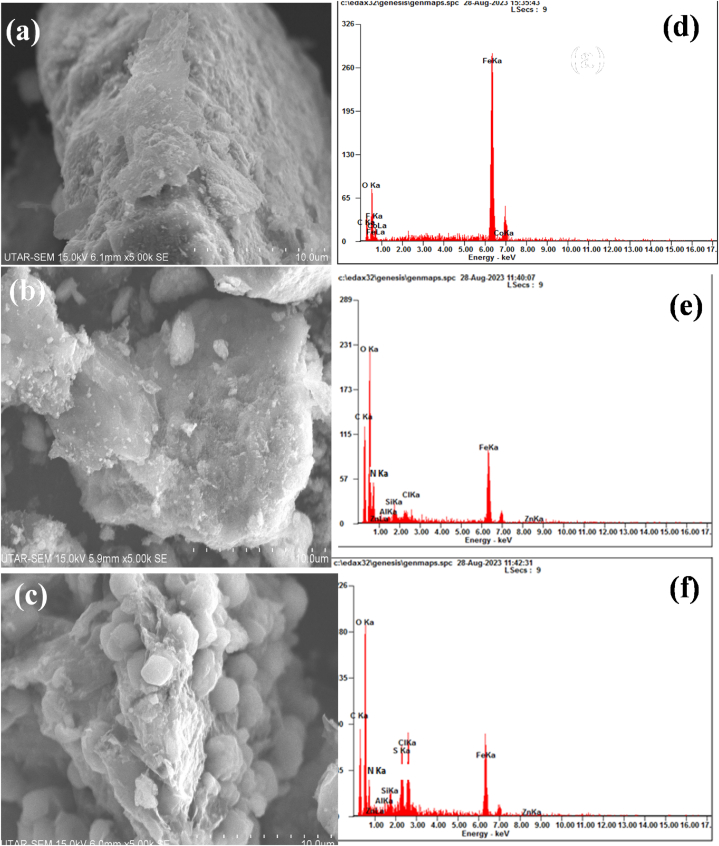
Surface morphology, (a) mGO, (b) mGO/CS and (c) mGO/PA nanocomposites and elemental composition, (d) mGO, (e) mGO/CS and (f) mGO/PA nanocomposites.

SEM and EDX analysis identified the morphology and composition of the mGO, mGO/CS and mGO/PA nanocomposites and the results are represented in Fig. 4. In Fig. 4(a–c), the images correspond to mGO, which showed the clear layers for GO. The broad surface of the GO sheets leads to the formation of wrinkles and folds in their appearance. Aggregation of mGO is reduced when its surface is grafted with the CS and PA polymers for the fabrication of nanocomposites as shown in Fig. 4(b and c). The EDX analysis in Fig. 4(a) and (b) and (c) validates the existence of Fe_3_O_4_ nanoparticles in all synthesized nanocomposites. The elemental composition of mGO was determined by EDX, which showed that it contains approximately 31 % oxygen, 13 % carbon, and around 51 % iron by weight in mGO. While in mGO/CS and mGO/PA nanocomposites, the % of iron present is between 25 and 39 % respectively. These results provide evidence for the successful synthesis and grafting of GO with Fe_3_O_4_ and polymers. The chemical interaction between graphene oxide with chitosan and polyaniline can involve a combination of hydrogen bonding, electrostatic, π-π stacking and in some cases, covalent bonding resulting in the formation of stable composite materials with unique properties [40]. GO, which acts as an electron acceptor/donor due to its π-conjugated structure, charge transfer interactions can occur between PANI and GO. This charge transfer can modify the electrical conductivity and electrochemical properties of the composite [41].

### 3.4 Box-Behnkin design

**Fig. 5:**
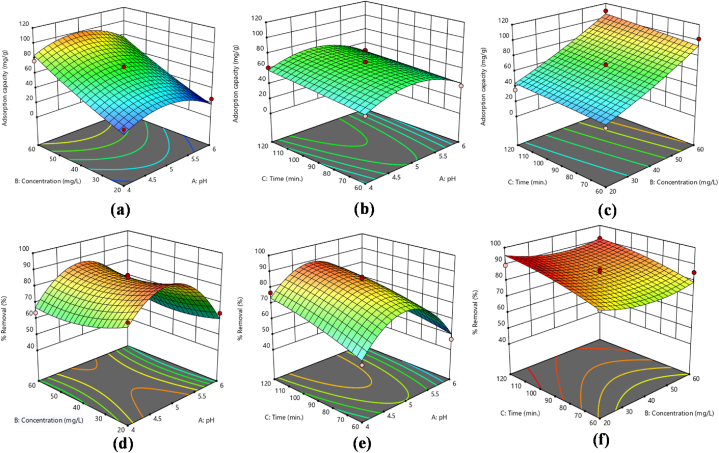
Response surface plots as a function of input variables, (a–c) adsorption capacity (d–f) % removal Pb ions onto mGO/CS nanocomposite.

**Fig. 6:**
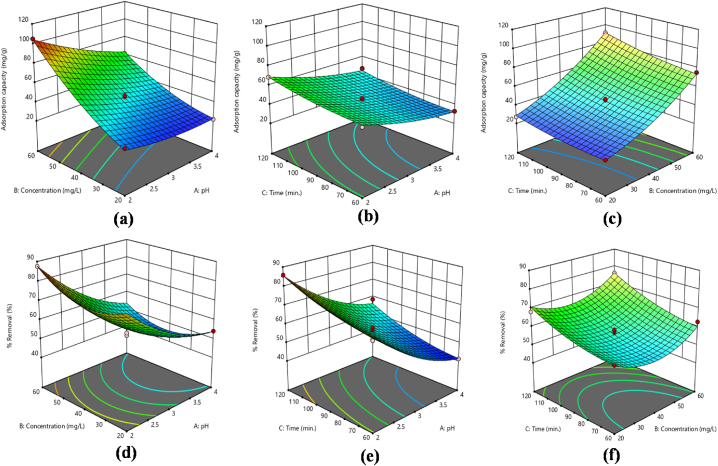
Response surface plots as a function of input variables, (a–c) adsorption capacity (d–f) % removal Cr ions onto mGO/CS nanocomposite.

**Fig. 7:**
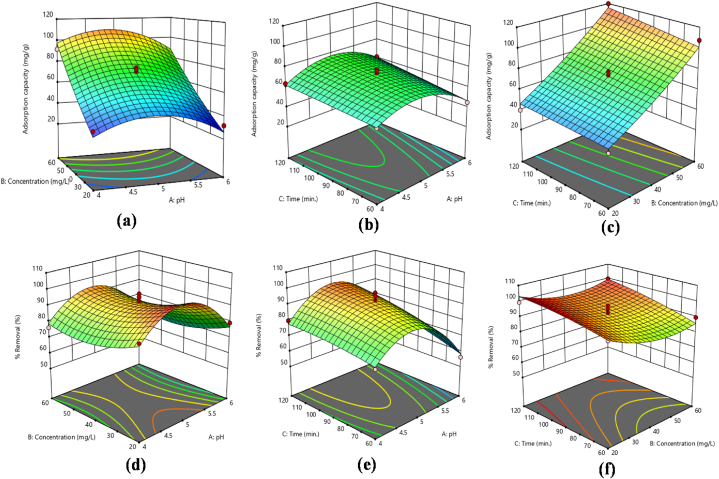
Fig. 8: Response surface plots as a function of input variables, (a–c) adsorption capacity (d–f) % removal of Pb ions onto mGO/PA nanocomposite.

**Fig. 8:**
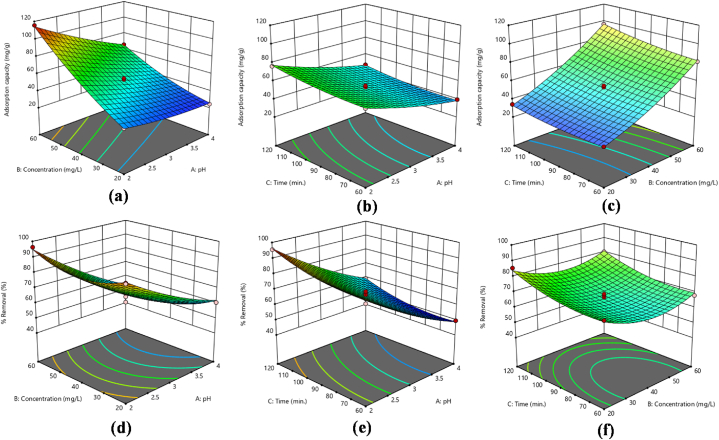
Response surface plots as a function of input variables, (a–c) adsorption capacity (d–f) % removal Cr ions onto mGO/PA nanocomposite.

Traditional optimization techniques typically involve altering one variable at a time while holding others constant. This single-variable approach often overlooks the complex interactions between variables and does not accurately reflect each parameter's impact on the outcome. Furthermore, traditional methods require a larger number of experiments to optimize various parameters, which increases the cost. While factorial experimental designs improve test validity, reliability, and repeatability and offer predictions with a specific level of statistical sensitivity. The BBD reduced the cost of experiments and yielded accurate and dependable experimental data. Renowned for its simple application as a factorial design, BBD is particularly valued for its predictive accuracy. This approach also provides information on how individual factors affect the response [24,30,42]. So, three variables with their three levels [−1, 0, +1] are studied in this project by applying BBD design. The variables comprised the initial concentration of metal ions, the pH of the solution and the contact time. A total of 17 runs were carried out, and the resulting data was analyzed statistically using Design Expert 13. The aim was to evaluate the influence of these variables on the sequestration of Cr(VI) and Pb(II) ions, with a focus on their effects on adsorption capacities (q_e_) and % removal. The results for Cr(VI) and Pb(II) ions are depicted in Table 2, Table 3, respectively. Additionally, Fig. 5(a–f), Fig. 6(a–f), Fig. 7(a–f) and Fig. 8(a–f) depict 3D surface plots illustrating the relationship between adsorption capacity and factors, as well as % removal and factors for Cr(VI) and Pb(II) ions, respectively.

#### 3.4.1 ANOVA analysis

To validate the effectiveness of the BBD analysis of variance (ANOVA), Table 4 presents the results. The model's F-value for Pb(II) using mGO/CS nanocomposite is 16.68, indicating its significance, with only a 0.06 % probability that this F-value could result from noise. The lack of fit F-value of 49.31 suggests statistical significance, with merely a 0.13 % chance that such a large lack of fit F-value could arise from noise. For Pb(II) ions adsorption by mGO/PA, the model's F-value is 23.72, indicating its significance, with only a 0.02 % probability of noise. With a 13.89 % probability that noise influenced the lack of fit F-value, the 3.31 value indicates that the lack of fit is not significant when compared to the pure error. According to ANOVA Table 4 for the Cr(VI) ions, the model F values are 145.46 and 202.44, implying that the model is significant for mGO/CS and mGO/PA, respectively, with only a 0.01 % chance that the F value could be large due to noise. The lack of fit F values is 1.36 and 0.48, indicating that lack of fit is not significant compared to the pure error, and there are 37.43 % and 71.32 % chances, respectively, that a lack of fit value could be large due to noise for mGO/CS and mGO/PA.P-values below 0.05 indicate that the model is significant for the data. For Pb(II) ions, A^2^ and B are significant model terms, while for Cr(VI) ions, A, B, C, AB, A^2^, and B^2^ are significant model terms as shown in Table 4.

**Table 4:** ANOVA for metal ions removal using mGO/CS and mGO/PA nanocomposite.

	Model terms Significant	Source	Sum of squares	df	Mean square	F-value	p-value
mGO/CS Pb(II)	A^2^, B	Model	8754.92	9	972.77	16.68	0.0006
		Residual	400.96	7	57.28		
		Lack of fit	390.41	3	130.14	49.31	0.0013
		Pure error	10.56	4	2.64		
		Cor total	9155.88	16			
mGO/PA Pb(II)	A^2^, B	Model	9780.61	9	1086.73	23.72	0.0002
		Residual	320.74	7	45.82		
		Lack of fit	228.66	3	76.22	3.31	0.1389
		Pure error	82.09	4	23.02		
		Cor total	10101.36	16			
mGO/CS Cr(VI)	A, B, C, AB, A^2^, B^2^	Model	8630.79	9	958.98	145.46	<0.0001
		Residual	46.15	7	6.59		
		Lack of fit	23.32	3	7.77	1.36	0.3743
		Pure error	22.83	4	5.71		
		Cor total	8676.94	16			
mGO/PA Cr(VI)	A, B, C, AB, A^2^, B^2^	Model	9398.68	9	1044.30	202.44	<0.0001
		Residual	36.11	7	5.16		
		Lack of fit	9.57	3	3.19	0.4807	0.7132
		Pure error	26.64	4	6.64		
		Cor total	9434.79				

#### 3.4.2 Modeling

For both coded and real factors, Design Expert offers two different types of equations. The response at specific levels of each variable can be predicted using the equation for actual factors since the levels of each variable are defined in the original units used for the corresponding factors. On the other hand, the coded equation can be used to determine the response for various factor levels, and the default setting does not include the higher levels of equations. The model equations for Pb(II) in both mGO/CS and mGO/PA are shown in equations (4), (5) in terms of coded factors [43,44]. Additionally, Equations (6), (7) represent the corresponding finalized equations for Cr(VI) in mGO/CS and mGO/PA, respectively.

(4) Pb(II) (q_e_) = 67.45–3.65 A + 29.30 B + 5.06 C + 0.2350 AB -1.48 AC + 1.17 BCE - 19.13 A^2^ + 2.93 B^2^ - 0.3110C^2^

(5) Pb(II) (q_e_) = 73.22–3.26 A + 32.29 B + 4.65 C–1.20 AB + 2.49 AC + 2.56 BCE - 16.12 A^2^ + 3.29 B^2^ - 0.2752C^2^

(6) Cr(VI) (q_e_) = 44.55–13.39 A + 28.50 B + 5.91 C–8.15 AB + 0.13 AC + 3 BCE + 3.68 A^2^ + 7.03 B^2^ + 2.67C^2^

(7) Cr(VI) (q_e_) = 52.57–14.91 A + 29.34 B + 4.56 C–9.42 AB - 0.39 AC + 2.46 BCE + 2.62 A^2^ + 5.3 B^2^ + 2.46C^2^

#### 3.4.3 Optimization of process variables

Optimization was conducted by applying a desirability approach, to achieve the maximum response. All three parameters were performed with the suggested values. The optimization results, as depicted in Table 5 of the Design Expert software, indicate that both the predicted and actual values of adsorption capacity (q_e_) and % removal of Pb(II) and Cr(VI) ions are within the 95 % confidence intervals (CI). This suggests that the models are valid with accuracy and account for the interaction among the variables.

**Table 5:** The optimal conditions suggested by Response Surface Methodology (RSM) and the experimental outcomes.

Adsorbent	Adsorbate	Optimal conditions			Predicted values		Actual values	
		pH	Conc. (ppm)	Time (min)	q_e_ (mg/g)	% R %	q_e_ (mg/g)	% R %
mGO/CS	Pb(II)	4.87	60	120	105.91	91.57	110.84	92.36
mGO/PA		4.96	60	120	115.78	99.5	118.44	98.7
mGO/CS	Cr(VI)	2	60	90	104.32	87.74	102.3	85.25
mGO/PA		2	60	90	114.16	94.95	111.7	93.08

### 3.5 Isotherm modeling

The relationship between the concentration of adsorbate in the solution and the concentration adsorbed onto the adsorbent surface under particular equilibrium conditions is clarified by the adsorption isotherms. These isotherms also provide a mechanism for explaining how the sorbate is distributed between the liquid and solid phases. Different types of isotherms, such as Freundlich and Langmuir, provide information about the interaction between the adsorbate and the surface of the adsorbent. By fitting experimental data to various isotherm models, we can determine the most suitable model that describes the adsorption behavior and gain an understanding of the driving forces behind the process [28,45]. Data were applied to the Freundlich (Eq. (8)) and Langmuir (Eq. (9)) isotherm models respectively,

(8) $${logq}_{e}={logK}_{F}+\frac{1}{n}\log\;C_{e}$$

(9) $$\frac{C_{e}}{q_{e}}=\frac{1}{q_{\max}K_{L}}+\frac{C_{e}}{q_{\max}}$$

In Eq. (8) K_F_ is the adsorption capacity (mg/g), adsorption intensity is represented by 1/n, q_*e*_ is the sorbate uptake (mg/g) and C_e_ is the concentration of a metal solution (mg/L) at equilibrium, while in Eq. (9) K_L_ represented the Langmuir isotherm constant. A good adsorbent is characterized by a higher value of q_max_ (maximum adsorption capacity) and an initially sharp isotherm [28].

Freundlich isotherm model showed the R^2^ values for adsorption of Pb(II) ions 0.9857 and 0.9755, while for Cr(VI) 0.9807 and 0.9298 against mGO/CS and mGO/PA respectively. The R^2^ values were higher in the Langmuir isotherm model compared to the Freundlich isotherm model for both Pb(II) and Cr(VI) ions adsorption. Langmuir isotherm had a good fitting to the experimental data for the adsorption of both metals’ ions due to a higher correlation coefficient (Table 6).

**Table 6:** Langmuir and Freundlich isotherms parameters for the removal of Pb(II) and Cr(VI) ions.

Adsorbate	Adsorbent	Freundlich isotherm			qe, Exp mg/g	Langmuir isotherm		
		K_F_ mg/g	1/n	*R*^2^		q_max_ mg/g	K_L_ mg/g	*R*^2^
**Pb(II)**	**mGO/CS**	7.771	0.8968	0.9857	145.28	456.62	0.1391	0.9876
	**mGO/PA**	2.761	1.130	0.9755	150.84	666.67	0.2845	0.9832
**Cr(VI)**	**mGO/CS**	21.82	0.627	0.9807	135.5	194.55	0.0561	0.9831
	**mGO/PA**	1.582	0.6154	0.9298	129.54	153.37	0.0848	0.9451

### 3.6 Kinetic modeling

The pseudo-first and second-order kinetic models were utilized to analyze the kinetic data for determining the adsorption rate. Both the pseudo-first-order (Eq. (10) and pseudo-second-order (Eq. (11) models have been widely employed to elucidate the adsorption mechanism [46,47].

(10) $$\log\left(q_{e}-q_{t}\right)=\log\;q_{e}-\;\left\{\frac{k_{1}}{2.303}t\right\}$$

(11) $$\frac{t}{q_{t}}=\frac{1}{k_{2}q_{e^{2}}}+\frac{t}{q_{t}}$$

**Fig. 9:**
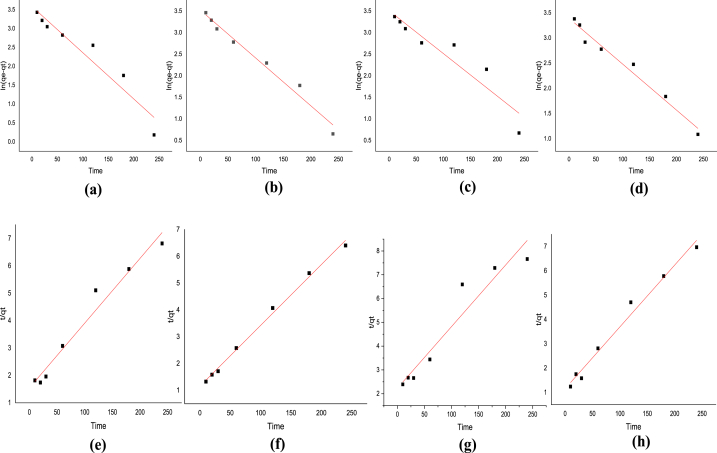
(a), (b), (c), and (d) Pseudo first-order kinetic graphs for Pb (II) ions by mGO/CS and mGO/PA and for Cr (VI) ions by mGO/CS and mGO/PA, respectively, while (e), (f),(g), and (h) pseudo second-order kinetic plots for Pb (II) ions by mGO/CS and mGO/PA, respectively, and for Cr (VI) ions by mGO/CS and mGO/PA nanocomposites, respectively.

In these equations, q_e_ represents the mass of metal adsorbed (mg/g), while q_t_ denotes the mass of metal at time t (min). The parameters k_1_ and k_2_ correspond to the first-order and second-order rate constants (mg/g min^−1^), respectively. Table 7 provides a comparison between the pseudo-first and second-order kinetic models for both metals. Fig. 9(a–h) shows the pseudo-first-order and pseudo-second-order kinetic plots for the representation of the correlation. The kinetic data suggest that the pseudo-second-order model, characterized by a higher regression coefficient (R^2^), exhibits good alignment with the data. This model assumes that adsorption may be the rate-limiting step and follows a second-order reaction mechanism, where the rate is proportional to the square of the adsorbate concentration. The favorable fit indicates that the experimental data closely matches the theoretical predictions of the pseudo-second-order model, implying that the process is likely controlled by chemisorption [28].

**Table 7:** Pseudo-second and pseudo-first-order kinetic model parameters for the removal of Pb(II) and Cr(VI) ions.

Adsorbate	Adsorbent	Pseudo-first-order kinetic model			q_e, Exp_ mg/g	Pseudo-second-order kinetic model		
		q_e_ mg/g	K_1,ads_ min^−1^	*R*^2^		q_e_ mg/g	K_2,ads_ mg/g	*R*^2^
**Pb(II)**	**mGO/CS**	36.12	5.08 x10^−5^	0.9222	40.12	42.37	0.00036	0.9681
	**mGO/PA**	33.89	4.6 x 10^−5^	0.9772	42.36	44.05	0.00044	0.9952
**Cr(VI)**	**mGO/CS**	33.32	4.1 x 10^−5^	0.8873	37.02	38.61	0.0.00030	0.9241
	**mGO/PA**	29.75	3.8 x 10^−5^	0.9734	36.81	39.33	0.00056	0.9839

### 3.7 Adsorption mechanism

**Fig. 10:**
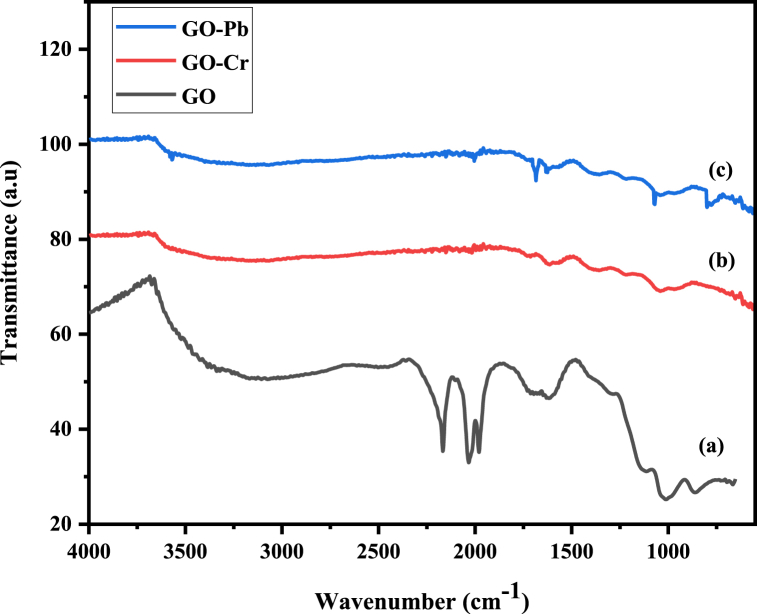
FTIR spectra of (a) GO (b) GO after Cr (VI) ions adsorption (c) GO after Pb (II) ions adsorption.

The results obtained from the pH investigation suggested that the optimal pH for the adsorption process was 2 for Cr(VI) ions and 5 for Pb(II) ions. In this context, the nanocomposite materials play a crucial role because they contain active groups NH_2_ and OH, which become protonated when exposed to acidic conditions. Consequently, the surface of nanocomposites became positively charged. While hexavalent chromium mainly exists as negatively charged HCrO_4_^1−^ in the acidic medium. This charge difference leads to a strong electrostatic attraction between the protonated groups of nanocomposites and the negatively charged Cr(VI) species, facilitating the adsorption of Cr(VI) onto both nanocomposites. This adsorption mechanism was observed in previous studies [19,31,48]. The FTIR spectra of the GO were analyzed before and after adsorption, as shown in Fig. 10(a–c). The FTIR spectra of GO after the adsorption of Cr(VI) and Pb(II) ions showed a noticeable decline in the vibrational intensity of the peaks at 3400 cm^−1^ and 1630 cm^−1^ compared to the peaks observed in GO alone. This decline strongly indicates that a chemical reaction occurred between the Cr(VI) and Pb(II) ions and the OH and carbon-containing functional groups present on the GO surface [49]. While mGO/PA and mGO/CS showed maximum removal of Pb(II) ions at pH 5 because this pH level optimizes the surface charge and ionization state of both the adsorbent and Pb(II) ions. At pH 5, the surface of the nanocomposites has a higher affinity for positively charged Pb^2+^, enhancing the adsorption efficiency. Additionally, at this pH, competition from hydrogen ions H^+^ for adsorption sites is minimized, allowing more Pb(II) ions to bind to the adsorbent. This specific pH also prevents Pb ions from precipitating as hydroxides, ensuring that they remain in a soluble form that is readily adsorbed [9,50]. Table 8 presents a comparison of various GO-based nanocomposites used for the efficient removal of Cr(VI) and Pb(II) ions from water.

**Table 8:** Comparison of maximum adsorption capacity for Pb(II) and Cr(VI) ions on different adsorbents based on GO nanocomposites.

Metal ions	Adsorbent	q_max_ (mg/g)	Reference
Cr(VI) ions	mGO/CS	87.74	Present study
	mGO/PA	111.7	Present study
	GO	1.22	[51]
	GO/Fe_3_O_4_	123.4	[52]
	Disodium ethylenediamine tetraacetate modified GO/CS composites(GEC)	86.17	[31]
	amino-functionalized magnetic graphenes	17.29	[53]
	GO-Manganese Ferrite	34.02	[54]
Pb(II) ions	mGO/CS	110.84	Present study
	mGO/PA	118.44	Present study
	Lignin/GO composite	71.32	[55]
	GO/ZnO/NiO	308.16	[56]
	GO/CS	312.28	[57]
	GO/polydopamine modified montmorillonite/carboxymethyl chitosan composite aerogel	465.12	[58]

## 4 Conclusion

The magnetic nanocomposite of graphene oxide with chitosan and polyaniline (mGO/CS, mGO/PA) was synthesized and characterized by FTIR, XRD, SEM and EDX techniques. The FTIR spectrum of GO showed peaks at 1630, 1050, 1618 and 1380 (cm^−1^) for C=O, C–O, C=C stretching and C–H bending vibrations respectively. In the mGO/CS nanocomposite, the peak at 1710 cm^−1^ indicated N–H bending and amide stretching, confirming the presence of CS. mGO/PA showed a peak at 3430 cm^−1^ for N–H, merged in the mGO/CS spectrum, and the peaks at 1620 and 1562 cm^−1^ for C=O, and C=C stretching of the quinoid ring, and 1415 cm^−1^ for C=C stretching of the benzenoid ring, that was absent in mGO/CS spectra. The average crystallite sizes of mGO, mGO/CS and mGO/PA nanocomposites determined using the Scherrer equation were to be 17, 25 and 23 nm, respectively. The adsorption parameters were optimized by employing the Box-Behnken design. The optimum pH for Pb(II) ions was determined to be 5, while for Cr(VI) ions, it was observed to be 2. mGO/CS and mGO/PA showed 92.36 and 98.7 (%) removal for Pb(II) and 85.25 and 93.08 (%) removal for Cr(VI) ions respectively under optimized conditions of solution pH, concentration of metal and contact time. The adsorption capacities were 110.84 and 118.44 (mg/g) for Pb(II), and 87.74 and 111.7 (mg/g) for Cr(VI) were obtained for mGO/CS and mGO/PA respectively. The precision and applicability of diverse models in illustrating the influence of adsorption parameters on both adsorption capacity and percentage removal were extensively examined. This examination was bolstered by high R-squared values (≥0.9) and statistically significant p-values below 0.05. The magnetic nanocomposite, consisting of graphene oxide, chitosan, and polyaniline, shows great promise across various domains due to its unique properties.

## Data availability

Data included in article/supp. Material/referenced in the article.

## CRediT authorship contribution statement

**Qaisar Manzoor:** Writing – original draft, Investigation. **Muhammad A. Farrukh:** Supervision, Conceptualization. **Muhammad T. Qamar:** Data curation. **Arfaa Sajid:** Formal analysis. **Samar A. Aldossari:** Software, Resources. **A. Manikandan:** Writing – review & editing, Writing – original draft. **Munawar Iqbal:** Writing – review & editing, Visualization.

## Declaration of competing interest

The authors declare that they have no known competing financial interests or personal relationships that could have appeared to influence the work reported in this paper.
